# Quantification of Total and Mutant Huntingtin Protein Levels in Biospecimens Using a Novel alphaLISA Assay

**DOI:** 10.1523/ENEURO.0234-18.2018

**Published:** 2018-10-10

**Authors:** Barbara Baldo, Muhammad Umar Sajjad, Rachel Y. Cheong, Julie Bigarreau, Ravi Vijayvargia, Catriona McLean, Anselme L. Perrier, Ihn Sik Seong, Glenda Halliday, Åsa Petersén, Deniz Kirik

**Affiliations:** 1 Translational Neuroendocrine Research Unit, Department of Experimental Medical Science, Lund University, Lund 22184, Sweden BMC D11; 2 Institut National De La Santé Et De La Recherche Médicale UMR861, I-Stem, AFM, Corbeil-Essonnes 91100, France; 3 UEVE UMR861, I-STEM, AFM, Corbeil-Essonnes 91100, France; 4 Center for Human Genetic Research, Massachusetts General Hospital, Boston, MA; 5 Department of Neurology, Harvard Medical School, Boston, MA; 6 Department of Pathology, Alfred Hospital, Melbourne, Victoria, Australia; 7 Sydney Medical School, Brain & Mind Centre, the University of Sydney, Camperdown, NSW 2050, UNSW Medicine & NeuRA, Kensington, NSW 2052, Australia; 8 Brain Repair and Imaging in Neural Systems, Department of Experimental Medical Science, Lund University, Lund 22184, Sweden BMC D11

**Keywords:** AlphaLISA, Huntingtin, Huntington’s disease, immunoassay, polyglutamines

## Abstract

The neurodegenerative Huntington’s disease (HD) is caused by a polyglutamine (polyQ) amplification in the huntingtin protein (HTT). Currently there is no effective therapy available for HD; however, several efforts are directed to develop and optimize HTT-lowering methods to improve HD phenotypes. To validate these approaches, there is an immediate need for reliable, sensitive, and easily accessible methods to quantify HTT expression. Using the AlphaLISA platform, we developed two novel sensitive and robust assays for quantification of HTT in biological samples using commercially available antibodies. The first, a polyQ-independent assay, measures the total pool of HTT, while the second, a polyQ-dependent assay, preferentially detects the mutant form of HTT. Using purified HTT protein standards and brain homogenates from an HD mouse model, we determine a lower limit of quantification of 1 and 3 pm and optimal reproducibility with CV values lower than 7% for intra- and 20% for interassay. In addition, we used the assays to quantify HTT in neural stem cells generated from patient-derived induced pluripotent stem cells *in vitro* and in human brain tissue lysates. Finally, we could detect changes in HTT levels in a mouse model where mutant HTT was conditionally deleted in neural tissue, verifying the potential to monitor the outcome of HTT-lowering strategies. This analytical platform is ideal for high-throughput screens and thus has an added value for the HD community as a tool to optimize novel therapeutic approaches aimed at modulating HTT protein levels.

## Significance Statement

The HTT-lowering approaches are widely investigated as possible therapies for HD. To support these strategies, there is a high need for reproducible and sensitive assays able to quantify HTT protein. In this study, we describe two robust and sensitive assays based on the AlphaLISA platform, which are able to measure either the pool of wild-type and mutant HTT, polyQ-independent assay, or preferentially mutant HTT, polyQ-dependent assay, in biological samples. These assays constitute a very valuable tool for HD research as they apply readily accessible antibodies and have a simple implementation. Furthermore, the HTT AlphaLISA assays are suitable for use in high-throughput studies and potentially could be multiplexed to monitor simultaneously different forms of HTT.

## Introduction

Huntington’s disease (HD) is a fatal neurodegenerative disorder caused by an expanded polyglutamine (polyQ) stretch at the amino (N)-terminal of the huntingtin protein (HTT). The disease is clinically manifested by cognitive, psychiatric, and motor symptoms and leads to premature death. There is no disease-modifying treatment available for HD patients at present.

HTT is ubiquitously expressed, but the mutant form of the protein causes toxicity mainly in the brain, leading to neuronal dysfunction and cell loss. The neuropathology in HD is characterized by neuronal death in the striatum, the cerebral cortex, and the hypothalamus (Vonsattel et al., 1985; Hedreen et al., 1991; Halliday et al., 1998; Gabery et al., 2010; Thu et al., 2010). Postmortem analysis of brains from HD patients show, in affected areas, nuclear and cytoplasmic inclusions enriched in the mutant HTT protein (DiFiglia et al., 1997). The mechanisms underlying mutant HTT toxicity are not fully understood; however, pathogenic steps are thought to include alterations of intracellular functions such as protein degradation, transcription, and axonal transport (Bates et al., 2015). Since the toxic gain of function of mutant HTT is likely to be critical for HD neuropathology, several pharmacological and gene therapy–based approaches are currently being explored with the aim of reducing mutant HTT levels (Harper et al., 2005; DiFiglia et al., 2007; Sarkar et al., 2007; Boudreau et al., 2009; Drouet et al., 2009; Baldo et al., 2012; Kordasiewicz et al., 2012; Yu et al., 2012). Several of these studies have relied on analysis of mRNA levels or protein analysis using semiquantitative Western blot. However, it is widely recognized that the ability to validate disease-modifying treatments would be significantly improved by the implementation of fully quantitative, sensitive, and specific assays.

In recent years, several immunoassays have been developed to monitor HTT in biological samples and explore the possibility to use HTT as a disease biomarker. The first assay applied the time-resolved fluorescence energy transfer (TR-FRET) technology to detect mutant HTT with antibodies targeting the N-terminal region of HTT and the polyQ tract (Weiss et al., 2009). Variations of this TR-FRET assay have then been implemented to measure total HTT [indiscriminating between wild-type (WT) and mutant HTT] while still targeting the N-terminal region of the protein (Weiss et al., 2011; Liang et al., 2014). With this assay, it was possible to obtain a lower detection limit (LDL) of 25 pm; however, its utility is hampered by the difficulty of detecting larger fragments of the protein, as the signal generated in the assay changes as a function of the proximity of the two antibodies bound on the target analyte.

A classic sandwich ELISA assay has also been developed for the detection of total HTT, using commercial antibodies specifically targeting a region outside the polyQ stretch with an LDL of 27 nm (2.7 fmol/well; Massai et al., 2013). Another ELISA-based assay developed for HTT detection applied the MesoScale Discovery platform, in which a measurable signal is generated after electrochemiluminescent amplification (Macdonald et al., 2014). MacDonald and colleagues developed multiple assays using different antibody pairs and detecting either total, mutant, or rodent HTT, showing an LDL in the low pm range. This method was also recently applied for the detection of HTT in leukocytes from HD patients (Hensman Moss et al., 2017). Two other studies have reported assays using either a single-molecule-counting (SMC) immunoassay or an immunoprecipitation assay combined with flow cytometry (Southwell et al., 2015; Wild et al., 2015) for detecting and quantifying soluble HTT in human cerebrospinal fluid (CSF), with a lower limit of quantification (LLoQ) in the fm range. Finally, an SMC assay has been developed recently to specifically measure phosphorylation at Threonine 3 of mutant HTT (Cariulo et al., 2017).

Here, we present two novel sensitive and robust assays for the quantification of HTT in biospecimens using commercially available antibodies. The assays use the AlphaLISA platform, a no-wash technology with a wide dynamic range and designed to be applied in high-throughput studies (Landeck et al., 2016). We describe a polyQ-independent assay for the quantification of total HTT (i.e., both WT and mutant form) and a second polyQ-dependent assay that preferentially detects the mutant form. We demonstrate that these assays have high sensitivity, provide a dynamic range, and are optimally reproducible in cells and animal models as well as in human brain tissue lysates.

## Material and Methods

### Antibodies and assay reagents

In this immunoassay, the signal is generated when two bead-coupled antibodies bind to the same analyte, thus coming in the desired proximity to each other. The subsequent excitation of donor beads allows for the release of singlet oxygen, leading to a chemiluminescent emission by the acceptor beads. Antibodies used in the AlphaLISA assay were obtained from commercial suppliers either as off-the-shelf products when possible or via custom orders to ensure that they were at 1 mg/ml concentration in PBS without any BSA, glycerol, or sodium azide present in the solution.

For the biotinylation of antibodies, 2 mg/ml NHS-ChromaLink-biotin (SoluLink) in PBS pH 7.4 was added to each antibody solution at a 30:1 molar ratio. The volume was adjusted to 100 μl with PBS pH 7.4 and incubated for 2 h at 23°C in the dark before filtering the mixture through Zeba spin columns (Thermo Fisher Scientific) at 1500 × *g* for 2 min to remove unbound biotin. This step was repeated 2–3 times by the addition of 300 μl of PBS on top of the resin and the tube centrifuged at 1500 × *g* for 1 min. The antibody concentration and biotinylation efficiency were measured using a NanoDrop2000 instrument (Thermo Fisher Scientific). Optical density (OD) values at 280, 354, and 450 nm were used to assess total protein concentration, biotin concentration, and presence of aggregates respectively. Biotinylated antibodies were stored at 4°C at a concentration of 500 nm in PBS with 0.05% NaN_3_, and 0.1% Tween 20.

Before the coupling of antibodies to beads, 1 mg of europium acceptor-beads (AlphaLISA Acceptor-beads, PerkinElmer) were washed with 50 μl PBS and centrifuged at 16,000 × *g* for 15 min. The beads were then resuspended in a 10:1 bead-to-antibody weight ratio (0.1 mg antibody). The volume was adjusted to 200 μl, with 0.13 m phosphate buffer pH 8.0. Next, 1.25 μl of 10% Tween 20 and 10 μl freshly prepared 25 mg/ml NaBH_3_CN in H_2_O were added (final concentrations of 0.06% and 1.18 mg/ml, respectively). The antibody-bead mix was then incubated for 48 h at 37°C. 10 μl freshly prepared 65 mg/mL CMO in 0.8 m NaOH were added to block unreacted sites and incubated for 1 h at 37°C. The antibody-bead mix was then centrifuged for 15 min at 16,000 × *g* and washed two times with 200 µl of 0.1 m Tris-HCl pH 8.0. The antibodies conjugated with acceptor beads were resuspended at 5 mg/mL concentration in storage buffer (200 µl PBS + 0.05% Proclin-300), vortexed, spun, sonicated for 5 min (Branson1210 sonication water bath), and stored at 4°C.

### Full-length human huntingtin purification

Purification of FLAG-tag huntingtin was conducted as previously described (Seong et al., 2010; Vijayvargia et al., 2016). Briefly, FLAG-tag huntingtin was expressed from pALHD(Q23,43,67) in the Baculovirus Expression system (Invitrogen). The Sf9 cell lysate, obtained by freezing/thawing in buffer A (50 mm Tris-HCl pH 8.0, 500 mm NaCl, and 5% glycerol) containing complete protease inhibitor cocktail and PhosSTOP phosphatase inhibitor cocktail (Roche Applied Science), was spun at 25,000 × *g* (2 h). The supernatant was incubated with M2 anti-FLAG beads (Sigma-Aldrich; 2 h, 4°C). The nonspecifically bound proteins were removed by washing extensively with buffer A. FLAG-huntingtin was eluted with buffer (50 mm Tris-HCl pH 8.0, 300 mm NaCl, 5% glycerol) containing 0.4 mg/ml FLAG peptide and loaded onto a calibrated Superose 6 10/300 column (GE Healthcare) equilibrated with 50 mm Tris-HCl pH 8.0 and 150 mm NaCl. FLAG-huntingtin eluted discretely and was estimated to be at least 90% pure by Coomassie staining. Quantitative assays of huntingtin proteins with varying polyglutamine sizes were performed with an equal amount of each protein, verified by DC protein assay (Bio-Rad) and R-250 Coomassie Blue staining of bands on 10% SDS PAGE, to control for potential differences in protein purity and amount. The molarity for all huntingtins was calculated using a molecular weight of 350 kDa deduced from the human cDNA sequence.

### PolyQ-independent and -dependent AlphaLISA assay protocols

Our objective was to develop and characterize two AlphaLISA assays for measuring human HTT, one aimed at detecting total HTT levels, i.e., indiscriminately both WT and the mutant protein (thus here termed the polyQ-independent HTT assay) and the other to specifically detect HTT with increased polyQ stretches (polyQ-dependent HTT assay). The assay development followed two steps of characterization. The first step was conducted to identify the most promising antibody pairs for the polyQ-dependent and polyQ-independent assays, using generic assay conditions. The second step aimed to optimize the best working conditions for the antibody pairs identified for each of the two assays.

The antibody screening step was run in 384-well plates (OptiPlate, PerkinElmer), and 5 µl (1 µg/well) of total protein from WT or BACHD brain homogenates were used as analyte matrix. HTT signal was measured with each antibody pair using 5 µl of 5 nm biotinylated antibody and 5 µl of 50 µg/ml of acceptor bead–conjugated antibody. The antibody-sample mix was incubated in the dark for 150 min at 23°C. 10 µl of 66.7 µg/ml donor beads were then added (AlphaScreen streptavidin-coated 547 Donor-beads, PerkinElmer) and incubated for 30 min at 23°C. The AlphaLISA signal was read with EnVision model 2104 (PerkinElmer). The signal-to-background ratio (S/B) of the different antibody pairs was then calculated. The antibodies used in this first screening are indicated in Table 1. The two antibody pairs showing the highest S/B ratio were selected for further characterization. After optimization of the protocol, the samples were run in triplicates in 384-well Alphaplate (PerkinElmer). The antibody-sample mix was incubated for 1 h in the dark after centrifugation for 10 s at 1 × *g*. Next, 15 µl donor bead (66.7 µg/ml) was added to each well and allowed a 30-min reaction time in the dark. The reading of the plate was then performed using EnVision (PerkinElmer) after excitation at 680 nm and using the AlphaScreen emission filter (570 nm).

**Table 1.** Antibodies used in the screening for the development of the AlphaLISA assay

				Host species			Detection	
Name	Clone	Provider	Cat #		Clonality	Epitope	H	M
Anti-huntingtin protein	1HU-4C8	Millipore	MAB2166	Mouse	Monoclonal	aa 414–503	Yes	Yes
Anti- polyglutamines	3B5H10	Sigma	P1874	Mouse	Monoclonal	polyglutamines	Yes	Yes
Huntingtin antibody	n.a.	Cell Signaling	CST2773	Rabbit	Polyclonal	residues surrounding Pro1220	Yes	Yes
Huntingtin	D7F7	Cell Signaling	CST5656	Rabbit	Monoclonal	n.a.	Yes	Yes
CHDI147	n.a.	Coriell	CH00149	Rabbit	Polyclonal	Mouse Htt aa 37–53 (proline-rich)		
CHDI146	n.a.	Coriell	CH00216	Rabbit	Polyclonal	Human Htt aa 54–70 (proline-rich)		
CHDI137	n.a.	Coriell	CH00146	Rabbit	Polyclonal	aa 4–19 (N-terminal)		
Huntingtin antibody (N-18)^1^	n.a.	Santa Cruz	sc-8767	Goat	Polyclonal	N-terminal (N-18)	Yes	Yes
Rat anti-huntingtin protein	mHD549	Millipore	MAB2174	Rat	Monoclonal	aa 549–679	Yes^2^	No

1 Discontinued by Santa Cruz.

2 Tested and confirmed in monkeys, tested in human.

The hook points for the two assays were determined with a serial dilution of the biotinylated antibody in the presence of a constant concentration of acceptor beads (50 µg/µl), donor beads (40 μg/ml), and analyte (1 µg/well of mouse brain lysate or 10 pm/well of purified HTT). Biotinylated CST 5656 antibody was serially diluted in acceptor-bead solution with either MAB2166 or P1874 using 3-fold dilution steps starting from a concentration of 10 nm and ending at 0.01 nM.

Standard curves were generated by a serial dilution of the stock of recombinant proteins (5 nM) 1:3 using assay buffer, resulting in 8 dilutions ranging between 5 nm and 1.5 pm. The first standard was used undiluted, thus in storage buffer (50 mm Tris HCl pH 8.0, 150 mm NaCl). Since the difference in polyQ implicates a difference in molecular weight, concentrations were converted to µg/µl before fitting the sigmoid standard curves in GraphPad Prism 7. Assay buffer was used as blank.

### Preparation and processing of biospecimens before use in the assay

For *in vitro* experiments, HEK293 cells were seeded in 6-well plates using culture medium (DMEM with 4500 mg/l d-glucose, non-essential amino acids (NEAA), 5% FBS, 1.9 mm l-glutamine, 40 µg/ml gentamicin) and transfected with full-length HTT expressing different polyQ stretches (23, 45, 73, 97, 145Q) using 6 µl Lipofectamine 2000 (Thermo Fisher Scientific) per well and 2 µg DNA. 24 h after transfection, the cells were harvested using 1% SDS lysis buffer (50 mm NaCl, 100 mm Tris-HCl pH 7.4, 1 mm EDTA, 1% SDS) containing protease and phosphatase inhibitors (Roche). Cells were sonicated 15× 1 s using a probe sonicator at 40 Hz (Sonics at Materials), the samples were incubated 10 min on ice, and centrifuged at 20,000 × *g* for 10 min at 10°C. Protein concentration was determined using the DC kit (Bio-Rad), following manufacturer instructions. The results are determined from 3 independent experiments.

Neural stem cell (NSC) cultures used in the short interference RNA (siRNA)-induced htt knockdown experiments were generated from human induced pluripotent stem cell (hiPSC) lines from a healthy control (i90-16, Tropel) and a disease case (HD-60, Coriel). Briefly, iPSCs were maintained on vitronectine (Invitrogen) matrix in StemMACS medium (Miltenyi). Cells were fed daily and manually passaged every 5–7 days. iPSC from passage 30 to 34 were used for NSC differentiation as previously described (Boissart et al., 2013). Briefly, commitment of hiPSC to the neural lineage was performed on dual SMAD inhibition using N2B27 medium [supplemented with XAV 939 (1 µm Tocris), LDN (0.1 µm Tocris) and SB431542 (20 µm; Sigma-Aldrich)]. At day 10, neural rosettes containing neuro-epithelial cells were manually collected and plated on poly-l-ornithine/laminin coating in N2B27 containing epidermal growth factor (EGF, 10 ng/mL, Peprotech) and FGF-2 (10 ng/mL, Peprotech). At confluence, cells were passaged using 0.05% trypsin (Invitrogen) and plated at the density of 50,000 cell/cm^2^. The NSC medium was changed every 2 days and passaged once or twice per week for no more than 20 passages.

For allele-specific knocking down of HTT, 5 million NSCs were used per condition and suspended in 100 µL Nucleofection solution (Lonza, Mouse Neural Stem Cells Nucleofector Kit). 5 µL of the siRNA of interest were added (Boissart et al., 2013). The mix was then transferred to a cuvette and electroporated with the Nucleofector 2b device. After transfection, 1 mL warm N2B27 medium was added to the cuvette. The transfected cells were directly transferred on poly-l-ornithine/laminin coating at the density of 25,000 cells/cm^2^. The experiment has been performed every other day 3 times. Samples were collected using trypsin and later used to perform AlphaLISA.

For detection of human mutated HTT protein in brain samples, male BACHD mice were obtained from Jackson Laboratory and crossed with FVB/N females to establish the BACHD colony. Nestin-Cre animals were produced as described earlier (Tronche et al., 1999) and crossed with the BACHD to obtain a Nestin-Cre × BACHD line. Animals were housed in groups of 5–7 per cage at a 12-h light/dark cycle with *ad libitum* access to normal chow diet. All animal procedures were performed in accordance with Lund University Animal Welfare and Ethics committee in the Lund Malmö region. Cull of the animals for the experiments in this paper was performed at P0 by decapitation (sex of the animals was not assessed). Half hemispheres of the brain and liver were fresh frozen and kept at –80°C until further processing. The tissue was lysed using 1% SDS lysis buffer (150 mm NaCl, 50 mm Tris base, 2 mm EDTA, 1% SDS) containing protease and phosphatase inhibitors. After sonication 15× 1 s (or until no more tissue residuals were visible) at 40 Hz using a probe sonicator (Sonics at Materials), the samples were incubated 10 min on ice and centrifuged at 20,000 × *g* for 10 min at 10°C. Protein concentration of the samples was measured using the DC protein kit (Bio-Rad). After lysis, the protein samples were stored at –80°C.

For detection of HTT in human postmortem brains, fresh frozen human brain tissue from the cerebral cortex, striatum, and cerebellum was dissected from HD cases and controls. The human postmortem tissue was obtained from the Sydney Brain Bank and Victoria Brain Bank Network in Australia, after approval of the project by the Sydney Brain Bank’s Scientific Committee (PID167). Demographic data are shown in Table 2. All individuals had given their informed consent before the donation of their brains, and the brain donor programs were approved by the Institutional Human Research ethics committee. The tissue was first lysed using 1:10 weight:volume 1% Triton lysis buffer (150 mm NaCl, 50 mm Tris base, 2 mm EDTA, 1% Triton) containing protease and phosphatase inhibitors (Roche), the samples were then briefly vortexed and sonicated 30× 1 s (or until no more tissue residuals were visible) at 40 Hz using a probe sonicator. After 10 min incubation on ice, the samples were centrifuged at 20,000 × *g* for 10 min at 4°C. The supernatant (cytoplasmic fraction) was transferred to a new tube while the pellet was washed two additional times with Triton lysis buffer. The two fractions obtained were collected in two new tubes. The remaining pellet was further lysed adding 1:5 weight:volume 1% SDS lysis buffer (150 mm NaCl, 50 mm Tris base, 2 mm EDTA, 1% SDS) containing protease and phosphatase inhibitors. After brief vortexing to dissolve the pellet, the samples were left 10 min on ice and then centrifuged 10 min at 20,000 × *g* at 21°C. The supernatant (nuclear fraction) was collected in a new tube for further analyses. The pellet was washed once more with SDS lysis buffer. Protein concentration of the samples was measured using the DC protein kit (Bio-Rad). Samples were stored at –80°C until further use.

**Table 2.** Demographic data for HD and control cases used for cortical, striatal and cerebellar analyses

Case	Age/sex	CAG	DD	Cause of death	PMD	Brain	Grade
HD1	68/m	44	13	Pneumonia	10	1184	3
HD2	69/f	42	20	Cardiorespiratory failure	2	1149	2
HD3	57/f	44	22	Pneumonia	22	800	4
HD4	61/m	43	17	HD end stage	17	1280	4
HD5	71/m	42	12	Myocardial infarct	41	1270	2
HD6	39/m	54	13	Cardiorespiratory failure	10	1047	4
HD7	39/f	46	11	HD end stage	36	680	3
HD8	58/m	46	11	HD end stage	32	1260	3
HD9	61/m	45	14	Sepsis	40	1500	4
HD10	62/m	43	12	HD end stage	22	1185	3
HD11	62/m	43	10	Pneumonia	24	1380	3
HD12	67/m	43	15	HD end stage	37	1200	2
HD13	67/m	45	15	Pneumonia	19	952	n.d.
HD14	71/m	40	10	Pneumonia	39	1150	3
HD15	72/m	43	33	Pneumonia	22	940	2
HD16	74/m	40	12	Cardiorespiratory failure	27	1475	2
HD17	77/m	41	20	Pneumonia	9	1018	4
C1	69/m			Pulmonary embolism	24	1290	
C2	67/f			Pulmonary embolism	26	1298	
C3	57/m			Ischemic heart disease	48	1372	
C4	73/m			Ischemic heart disease	43	1532	
C5	64/m			Ischemic heart disease	32	1335	
C6	64/m			Ischemic heart disease	24	1492	
C7	66/f			Metastatic carcinoma	43	1233	
C8	69/m			Ischemic heart disease	34	1240	
C9	76/m			Aortic aneurysm	46	1459	
C10	78/m			Ischemic heart disease	46	1471	

### Caspase-6 in vitro assay

Purified standard HTT protein expressing 32Q was diluted 1:7 in caspase reaction buffer (50 mm HEPES, pH 7.4, NaCl 100 mM, 0.5% CHAPS, 1 mm EDTA, and 10 mm DTT) and incubated with 400 U of active human recombinant caspase-6 (Enzo Life Sciences, BML-SE170-5000) for 2 h at 37°C. Then, 0.5% SDS was added to inactivate the caspase-6 before performing the HTT assays. Percentage of variation in the AlphaLISA signal was calculated based on a standard curve generated with the 32Q purified HTT.

### Data and statistical analysis

S/B was calculated as signal (AlphaLISA counts) of the analyte divided by the signal of blank. The LLoQ was calculated as mean of 3 blank replicates + 6 * SD. GraphPad Prism7 was used for calculating the fitting of the values measured for the HTT standard curves over a sigmoid weighted curve and corresponding log-transformed HTT concentration values. Blanks were set to 1 × 10^–25^ mg/ml.

Intra-assay reproducibility was determined by calculating the percentage of variation for 6 independent samples from the mean of three experiments repeated the same day. Interassay reproducibility was determined by calculating the percentage of variation for one standard curve (*n* = 8) between the mean of three experiments repeated in three different days. Relative standard deviation was calculated for matching samples run on the same day or on different days.

Z′ values are calculated as follows:

$$\text{Z}’=\text{1}-\frac{\text{3x}\left({\text{STD signal}}_{\text{analyte}}+{\text{STDsignal}}_{\text{blank}}\right)}{{\text{Mean signal}}_{\text{analyte}}-{\text{Mean signal}}_{\text{blank}}}$$

Data are shown as mean ± SEM, unless otherwise specified. GraphPad Prism7 was used for statistical analysis. Significance was considered for *p* < 0.05. After verifying normal distribution using Kolmogorov–Smirnov test, one-way ANOVA followed by a Tukey’s *post hoc* test or Kruskal–Wallis followed by Dunn’s *post hoc* test were performed. For multifactor analysis of human postmortem tissue, two-way ANOVA with Tukey’s multiple comparison test was used.

## Results

### Screening for antibodies suitable for HTT AlphaLISA assay

**Figure 1.**
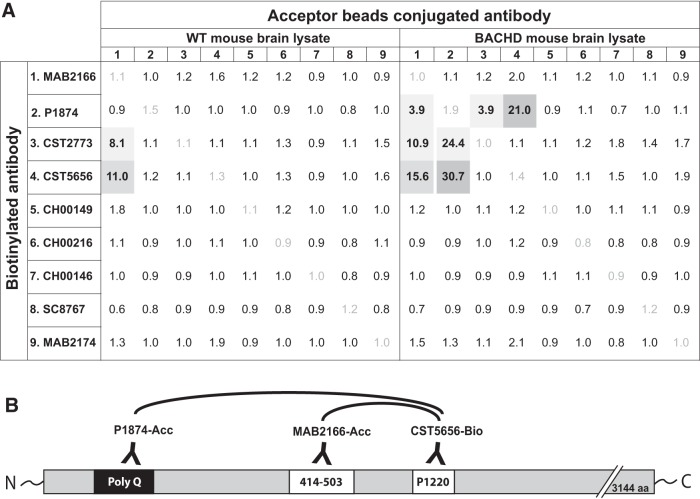
Antibody screening for the HTT AlphaLISA assay. Signal-to-background ratios obtained in the screening steps for the polyQ-dependent and -independent AlphaLISA assays are presented for each pair of antibodies tested. Antibody combinations producing a signal-to-background ratio different from 1 are represented in gray, with selected antibody combinations for the HTT AlphaLISA in dark gray (***A***). Note that these experiments were conducted without optimization of the assay conditions for each antibody pair. Schematic representation of the antibodies selected for the AlphaLISA assay and their binding sites on the HTT protein. The biotinylated antibody (CST5656) is combined with Acceptor bead-conjugated antibodies: MAB2166 for the polyQ-independent assay and P1874 for the polyQ-dependent assay (***B***).

In this study, we report two assays developed for the quantification of HTT protein in biospecimens either independent of polyQ length (readout of total HTT levels) or in a polyQ-dependent manner (readout of the mutant form of the protein). To this end, we used the AlphaLISA platform from PerkinElmer, which has previously been used for the detection of alpha-synuclein protein (Landeck et al., 2016). As a first step, we aimed to find the best antibody combinations for the two assays. In the first screening step, we chose several commercially available antibodies (see Table 1) and determined the signal-to-background ratio in the AlphaLISA platform. In this step, the antibodies were used in biotinylated or acceptor bead–conjugated forms using the BACHD and WT mouse brain tissue homogenates as source for the target analyte (Fig. 1*A*). Biotinylation of CST5656 (CST5656-bio), which has its binding epitope around Proline 1220, combined with acceptor beads–conjugated MAB2166 (MAB2166-Acc; epitope at amino acids 414–503) produced S/B ratios of 11.0 and 15.6 in WT lysate and BACHD lysate, respectively, suggesting that these antibodies could be used for the polyQ-independent assay (Fig. 1*B*). The use of biotinylated CST5656 produced a good signal (S/B: 30.7) in BACHD samples when combined with Acceptor bead–conjugated p1874 (P1874-Acc), targeting the polyQ region encoded in Exon1 (Fig. 1*B*), while WT mouse brain gave no detectable signal (S/B: 1.2). Hence, this second antibody combination was selected for the polyQ-dependent assay.

### Assay optimization

**Figure 2.**
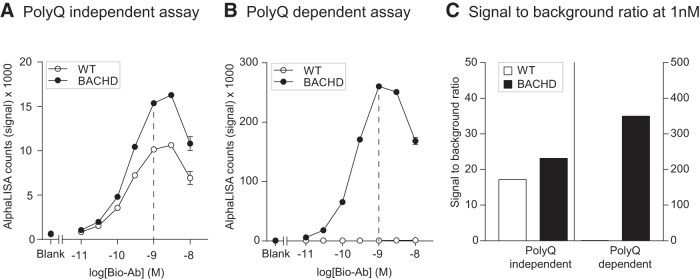
Hook point analyses of polyQ-dependent and -independent AlphaLISA assays in mouse brain lysates. The hook point was determined using serial dilutions of the biotinylated Ab (CST5656) in combination with the Acceptor-bead conjugated antibody MAB2166 for the polyQ-independent assay (***A***) and P1874 for the polyQ-dependent assay (***B***) to detect HTT in protein homogenates from brain tissue of BACHD or WT mice (1 µg/µl). Most optimal assay conditions are indicated with a dashed line, at 1 nm of biotinylated antibody. Signal-to-background ratio has been calculated for 1 nm concentration of the biotinylated antibody (***C***). Data in ***A*** and ***B*** are presented as mean ± SEM.

After determining the most suitable antibody combinations for the two assays, we optimized the working conditions of the assay by performing a hook point analysis. This analysis aims to determine the optimal concentration of the biotinylated antibody for obtaining a high S/B ratio without the unnecessary use of antibodies. A series of concentrations ranging from 0.01 to 10 nm were tested using CST5656-bio in combination with constant amount of MAB2166-Acc or P1874-Acc. As analyte, we used a constant concentration of either WT or BACHD brain homogenates, in the two respective assays. We found that both assays performed in an optimal fashion when 1 nm CST5656-bio was used (Fig. 2*A*, *B*). Using the polyQ-independent assay, we found a higher signal in BACHD brain homogenate compared to WT (Fig. 2*A*). This result was expected since mutant HTT is overexpressed compared to endogenous protein in the BACHD mouse (Gray et al., 2008). Also, the polyQ-dependent assay did not produce any signal in the WT brain homogenate (Fig. 2*B*). At the hook point, the S/B ratio for the polyQ-independent assay at 1 µg/µl protein concentration was 17.2 in WT tissue and 23.2 in BACHD tissue, while in the polyQ-dependent assay the corresponding values were 1.1 and 349.8, respectively (Fig. 2*C*).

**Figure 3.**
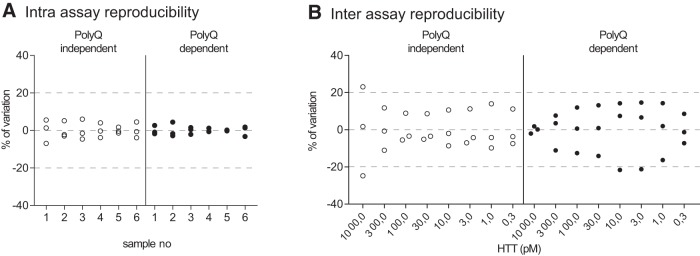
Intra- and interassay reproducibility. Intra-assay reproducibility for the polyQ-independent and -dependent AlphaLISA assays were determined using six independent samples of protein homogenates from brain tissue of BACHD mice (0.1 µg/well), measured on three different plates on the same day. Percentage of variation is reported (***A***). Interassay reproducibility for the same assays were determined using 8 dilutions of purified HTT with 23Q and 43Q, respectively, measured on three different days. Percentage of variation is reported (***B***).

We then aimed to verify the intra- and interassay variations for both assays. Intra-assay reproducibility was calculated using six independent samples measured on three different plates on the same day. All the samples for both polyQ-independent and polyQ-dependent assay had a 0.3%–4.6% percentage of variation from their mean, thus conforming to industry standards (Fig. 3*A*). Interassay reproducibility was calculated using 8-dilution series of purified HTT protein measured on three plates from three different days. For the polyQ-independent assay, all measurements resulted in a <20% percentage of variation from the mean, with the exception of the highest concentration. Of note, since the samples for this data point are undiluted and thus have a different buffer than the others, the variation might be influenced by the buffer matrix on the signal. For the polyQ-dependent assay, all the measurements resulted in a <20% percentage of variation from the mean, with the exception of two of the replicates (21.7% and 21.2%; Fig. 3*B*). Finally, the Z′ values calculated using 18 standard replicates and 18 blank replicates were 0.74 for the polyQ-independent assay and 0.84 for the polyQ-dependent assay.

### Increased signal detected with higher polyQ

**Figure 4.**
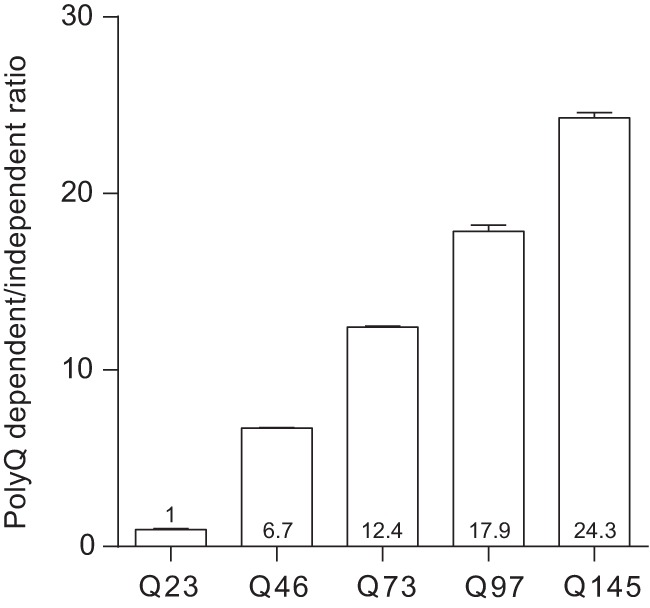
PolyQ-dependent signal increases with longer glutamine stretches. HEK cells were transiently transfected with full-length HTT expressing different polyQ lengths. The ratio between polyQ-dependent and -independent assay signal was calculated as average of the ratio in three different experiments where the average values are indicated in each bar for ease of reading. Data are presented as mean ± SEM.

After having confirmed the robustness of the assay and the best working conditions, we aimed to investigate whether the signal derived from the polyQ-dependent assay would significantly increase with increasing polyQ length of the HTT analyte. To this end, we used HEK293 cells transfected with human full-length HTT with different polyQ stretches (23, 45, 73, 97, 145Q). The cell lysates were analyzed with both the polyQ-independent and -dependent assays. A ratio calculated between the AlphaLISA readouts from the polyQ-dependent and -independent assays demonstrated that a higher number of glutamine repeats results in an increase in the signal (Fig. 4). This data suggested that to analyze and quantify the signal deriving from different polyQ stretches, the use of appropriate protein standards with similar polyQ stretch is required.

### Analyses of purified human HTT standards

**Figure 5.**
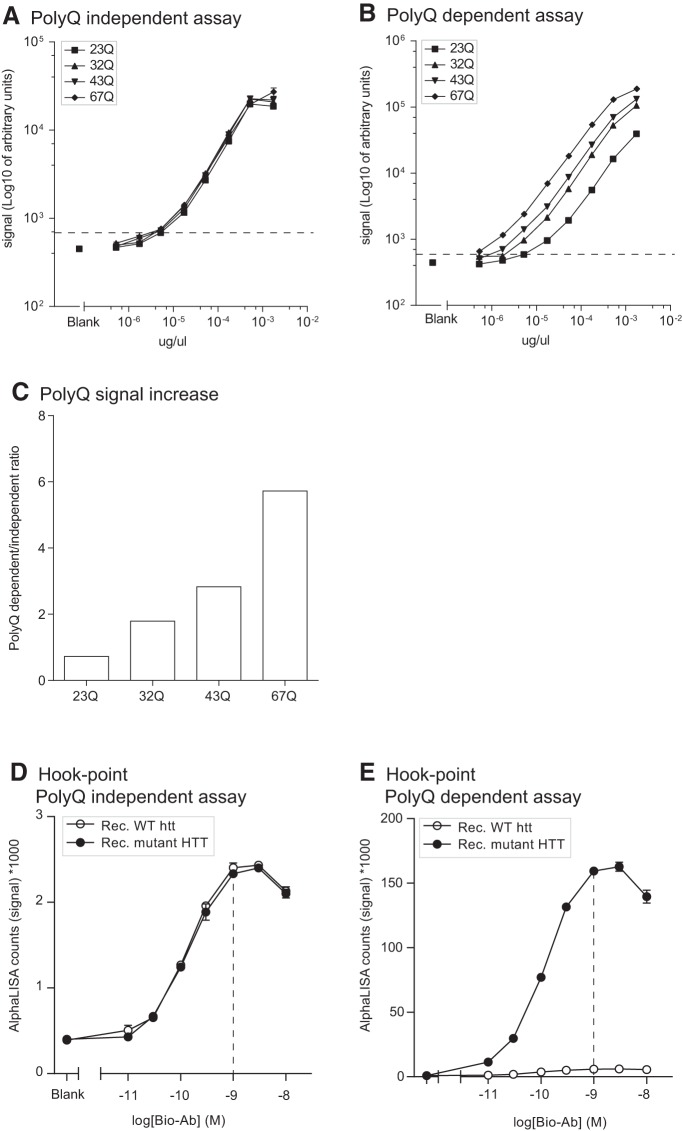
Purified HTT protein standards. Serial dilutions of full-length purified human HTT protein expressing different polyQ were used to generate standard curves with the polyQ-dependent and -independent assay (***A***, ***B***). Lower limit of quantification (LLoQ) was 3 pm per well for both assays and it is indicated by a dashed line on the graphs. polyQ-dependent increase in signal intensity is displayed as the ratio of the raw AlphaLISA counts in polyQ-dependent/-independent assay at 100 pm in each well (***C***). Hook points were calculated using serial dilutions of the biotinylated CST5656 in the presence of constant Acceptor beads-conjugated antibody and purified HTT protein with 23 or 78Q (***D***, ***E***). Best assay conditions are indicated with a dashed line at 1 nm of the biotinylated antibody. Data in ***A***, ***B***, ***D***, and ***E*** are presented as mean ± SEM.

The findings above in cell lines overexpressing transgenic human mutant HTT with increasing lengths of CAG repeats led us to explore the signal measured with both assays using standard curves generated with purified full-length human HTT expressing 23, 32, 43, or 67Q. This step was important in establishing the proper calibrators that are required for experiments in which the assay would be incorporated as a tool. We found that the polyQ-independent assay produced comparable signals between standards at the same concentration but with different polyQ length (Fig. 5*A*). On the contrary, and as illustrated above in cell lines, the polyQ-dependent assay showed increased signal for the same standards where the signal was increased with increasing polyQ length (Fig. 5*B*). At 100 pm analyte concentration and using the full-length human HTT with 23Q as the reference standard, we found that the ratio between polyQ-dependent and -independent signal was as much as 6-fold higher with 67Q HTT and that the signal change was proportional to the increase in polyQ length, i.e., 43Q HTT gave 3-fold change in signal (Fig. 5*C*). Furthermore, using these full-length protein standards, we found that the LLoQ for both assays was 3 pm. Finally, hook points generated using constant concentration of purified HTT proteins with 23Q or 78Q confirmed that the optimal assay conditions are obtained in the presence of 1 nm of biotinylated antibody (Fig. 5*D*, *E*).

### HTT detection and quantification in human postmortem brain tissue

**Figure 6.**
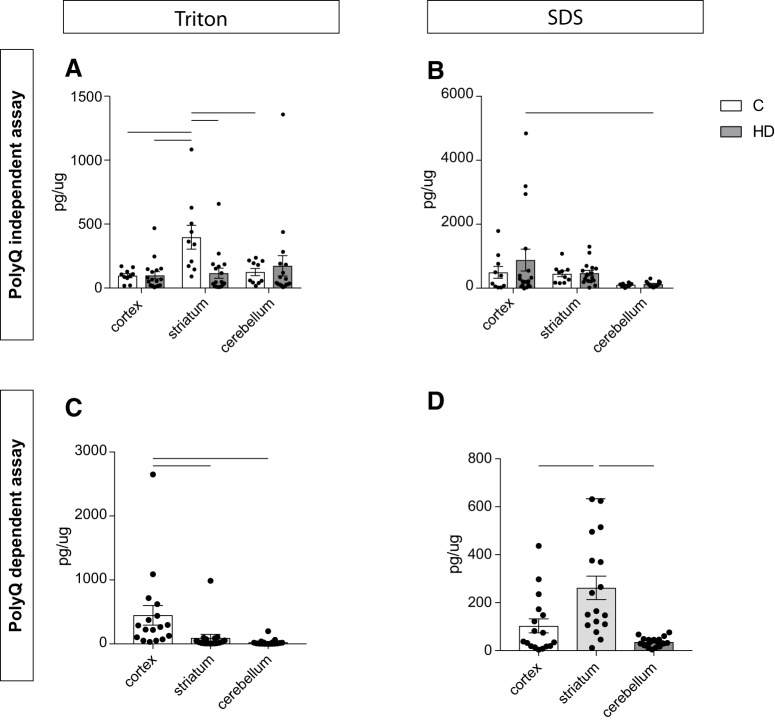
HTT detection in human postmortem tissue. Human postmortem tissue from cerebral cortex, striatum, and cerebellum was processed using the polyQ-independent (***A***, ***B***) and -dependent (***C***, ***D***) assays in cytoplasmic (Triton; ***A***, ***C***) and nuclear (SDS; ***B***, ***D***) fractions. In ***A*** and ***B***, statistical analysis has been performed using two-way ANOVA with Tukey’s multiple comparisons test (in ***A***: cortex C versus striatum C, *p* = 0.0211, cortex HD versus striatum C, *p* = 0.007, striatum C versus striatum HD, *p* = 0.0129, striatum C versus cerebellum C, *p* = 0.0479; in B: cortex HD versus cerebellum HD, *p* = 0.0329). In ***C*** and ***D***, statistical analysis has been performed using Kruskal–Wallis with Dunn’s *post hoc* test after verifying normal distribution using Kolmogorov–Smirnov test (in ***C***: cortex versus striatum, *p* = 0.0025, cortex versus cerebellum, *p* < 0.0001; in ***D***: cortex versus striatum, *p* = 0.0141, striatum versus cerebellum, *p* < 0.0001). The data are presented as mean ± SEM.

The two newly developed assays were then applied on extracts of frozen human postmortem tissue of cerebral cortex, striatum, and cerebellum from 10 healthy controls and 17 HD cases (Table 2). Our aim was to investigate whether different brain regions from clinical material display differences in the expression of human HTT proteins. The samples were lysed using a fractionation protocol applying a lysis step first aimed at extracting the cytoplasmic fraction (using Triton X-100) followed by two extra washing steps in the same buffer to remove all residual detergent-soluble proteins from the pellet. A second lysis step was then performed using SDS containing buffer to extract the nuclear proteins (note that this lysis step would also solubilize at least in part aggregated cytoplasmic proteins should they exist in the sample). The fractions were analyzed with both assays, and the AlphaLISA signal was quantified using appropriate standard curves. When looking at the polyQ-independent assay in the Triton-X fraction, we detected a significant difference in HTT expression between HD cases and controls in the striatal samples. The HTT protein levels recovered from the control cases were higher than the levels detected in HD cases (Fig. 6*A*). In addition, striatal samples in control cases presented with higher levels of HTT compared to cortical samples and cerebellar samples from the same individuals (Fig. 6*A*). In the SDS fraction, we could not detect any difference between HD cases and controls, suggesting that the whole nuclear fraction of HTT was unaltered (Fig. 6*B*). When looking at the polyQ-dependent assay in HD cases, in the Triton X-100 fraction we could detect higher levels of mutant HTT in cortex compared to the other two brain regions (Fig. 6*C*), while striata had increased levels of mutant HTT in the SDS fraction (Fig. 6*D*).

### Decrease in AlphaLISA signal on modulation of mutant HTT in vivo and in vitro

**Figure 7.**
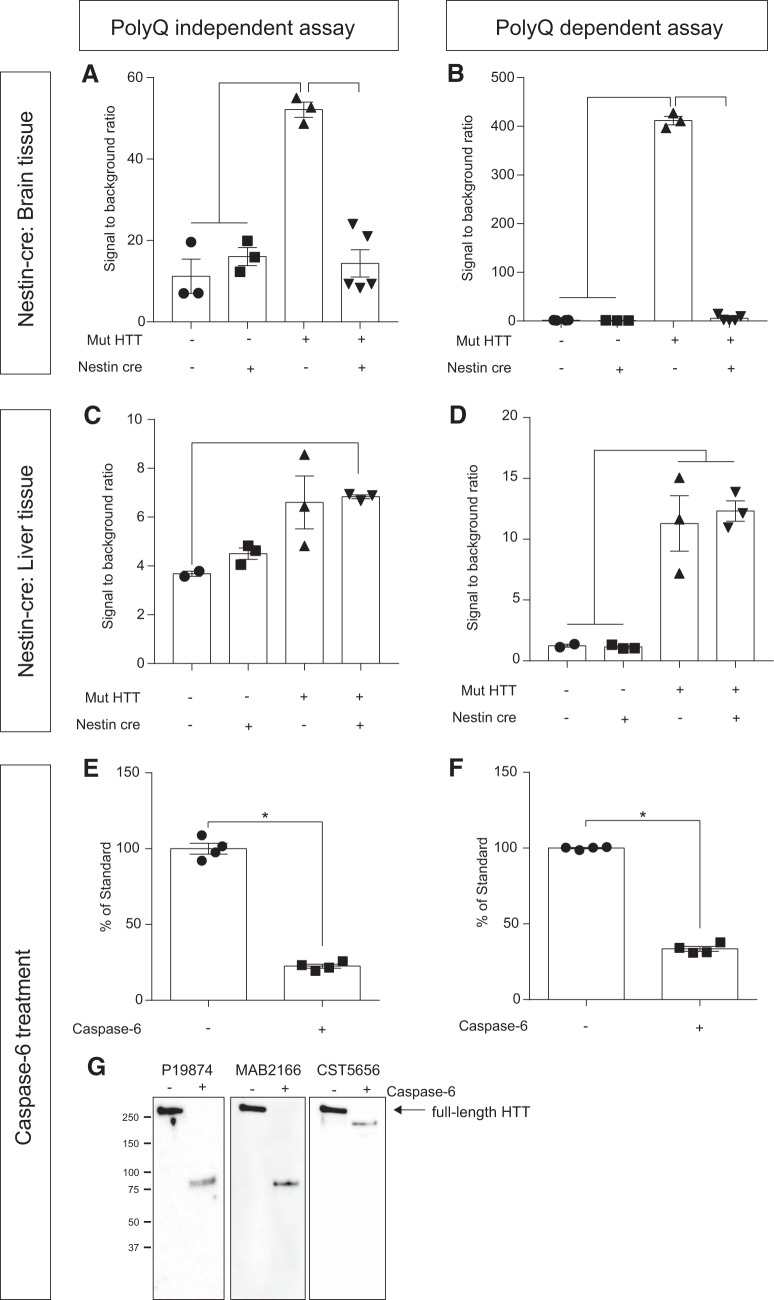
AlphaLISA signal changes on mutant HTT deletion in mice. BACHD mice have been crossed with Nestin-Cre mice, allowing Cre-dependent excision of mutant HTT from all neurons. Brain tissue lysates from BACHD × Nestin-Cre mice were analyzed with the polyQ-independent (***A***) and -dependent (***B***) assay and reported as signal-to-background ratios. HTT levels in the BACHD group were significantly different from the levels in the two WT groups as well as in the BACHD × Nestin-Cre mice in both the polyQ-independent and polyQ-dependent assay, indicating that the AlphaLISA signal is sensitive to genetic manipulations of HTT levels (one-way ANOVA with Tukey’s *post hoc* test BACHD in the polyQ-independent assay: *F*(3,10) = 30.46, *p* < 0.0001; BACHD in the polyQ-dependent assay: *F*(3,11) = 2410, *p* < 0.0001). As a control, liver tissue lysates from BACHD × Nestin-Cre mice were analyzed with the polyQ-independent (***C***) and -dependent (***D***) assay. In these samples, the polyQ-independent assay showed increased HTT levels in BACHD-NestinCre compared to WT (one-way ANOVA with Tukey’s *post hoc* test *F*(3,7) = 5.832, *p* = 0.0256; ***C***). In the polyQ-dependent assay, HTT levels in both BACHD and BACHD-NestinCre samples were statistically different from HTT levels in WT and NestinCre mice (one-way ANOVA with Tukey’s *post hoc* test *F*(3,7) = 20.36, *p* = 0.0008), but not different between each other (***D***). Changes in the AlphaLISA signal on treatment of purified HTT expressing 32Q with active caspase-6 were measured with the polyQ-independent (***E***) and -dependent (***F***) assay. An aliquot from the same reaction was processed for Western blot analysis using the same antibodies as in the assays (***G***) and shows HTT proteolytic fragmentation. The significant decrease of full-length HTT in the AlphaLISA signal in samples treated with caspase-6 was verified using students *t* test. Data are presented as mean ± SEM.

Next, we investigated whether the present assay could be used to measure changes in HTT levels, e.g., as a tool to assess the efficacy of therapeutic strategies aiming at HTT lowering. To this end, as a first step, we crossed BACHD mice with Nestin-Cre animals, thus allowing for Cre-recombinase–dependent deletion of mutant HTT in neural tissue globally. Protein homogenates from brain and liver tissue were obtained from four genotypes—WT animals, WT animals expressing Cre, BACHD animals, and BACHD animals expressing Cre—and samples were analyzed using the two assays. We expected to find that both assays would reveal a decrease in signal when HTT is deleted from the brain and found that the Nestin-Cre deletion reduced the polyQ-independent signal essentially to the same level as in WT animals, which is a reflection of the presence of mouse WT protein in the background (Fig. 7*A*). Furthermore, the polyQ-dependent assay showed essentially a complete loss of HTT protein bringing the signal below LLoQ (Fig. 7*B*). We used liver samples as a nonneural control tissue and as expected found no changes in total or mutant HTT levels in samples from BACHD mice expressing Cre under the nestin promoter compared with the BACHD mice lacking the Cre expression (Fig. 7*C*, *D*).

Several proteases and caspases have been identified to cleave the HTT protein at specific epitopes, inducing the formation of fragments. In particular, caspase-6 has been shown to cut HTT at amino acid (aa) 586 and be associated with behavioral and neuropathological features in HD (Graham et al., 2006; Pouladi et al., 2009; Graham et al., 2010). We therefore argued that detecting changes in HTT signals in the presence of short HTT fragments could be important in studying the effect of disease-modifying approaches. HTT assays developed so far have been implemented by using antibodies targeting the N-terminal part of the protein and as such are not suitable for identifying changes occurring in response to HTT fragmentation. Our assays are designed to target a longer portion of the HTT protein, and hence changes in the fragmentation of the protein would be reflected as changes in signal. The 586-aa residue where caspase-6 cleaves is located between the Acceptor-Ab and the biotinylated one, thus making the oxygen transfer improbable if the protein is cleaved at this residue. To verify this, we induced HTT-mediated caspase-6 cleavage *in vitro* before analyses with the AlphaLISA assays. After a 2-h incubation of purified HTT with 32Q in the presence of active caspase-6, we observed a reduction of AlphaLISA signal with both the polyQ-independent and -dependent assay, of 78% and 67%, respectively (Fig. 7*E*, *F*) and the detection of the HTT cleavage product using the antibodies in the AlphaLISA in Western blot (Fig. 7*G*).

**Figure 8.**
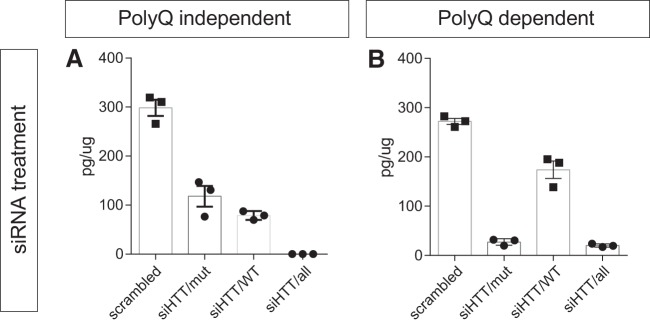
AlphaLISA signal changes on silencing of HTT. Neural stem cells generated from patient derived induced pluripotent stem cells were transfected with three different siRNAs targeting the mutant allele (siHTT/mut), the wild-type allele (siHTT/WT), or both (siHTT/all). Effects of HTT silencing were measured with the polyQ-independent (***A***) and dependent (***B***) assays. Data are presented as mean ± SEM.

Changes in the AlphaLISA signal can also be detected when HTT levels are modulated in cell culture models using siRNA (Fig. 8). Neural stem cells (NSCs) derived from patient-specific human induced pluripotent stem cells with 60 CAG repeats were treated with siRNAs targeting single nucleotide polymorphism (SNP) present in the HTT gene to selectively target the disease HTT isoform (siHTT/mut), the wild-type allele (siHTT/WT), or both alleles (siHTT/all) as previously described in Drouet et al. (2014) and Lopes et al. (2016). A scrambled siRNA sequence was used as control for the transfection. Quantification of the HTT levels in these samples using the polyQ-independent assay showed a reduction of 60% using HTT/mut, 74% using HTT/WT, and a complete loss of signal after administration of HTT/all (Fig. 8*A*). In the polyQ-dependent assay, the reduction was of 90% and 93% after treatment with HTT/mut and HTT/all, while we found a 37% reduction in the HTT/WT group.

## Discussion

A number of therapeutic approaches for Huntington’s disease aimed at reducing the levels of mutant HTT and improving clinical features are currently being tested (Aronin and DiFiglia, 2014; Wild and Tabrizi, 2017). To measure the extent of HTT reduction and to evaluate the impact of reducing the WT and mutant form of the protein, methods are needed that are quantitative, robust, and sensitive.

We have developed two assays to quantify either total HTT, independently of polyQ, or mutant HTT, depending on the polyQ length, in biospecimens with comparable or improved characteristics to previously designed bioassays. The no-wash bead-based AlphaLISA assay we used for quantification of HTT proteins have shown excellent reproducibility and sensitivity not only in protein standards but also in cell extracts from *in vitro* preparations, tissue specimens from mouse models, as well as human postmortem brain samples. Importantly, the use of commercially available antibodies for their development increases this assay’s value as a widely implementable analytical tool. Contrasting to most of the previously developed assays, which were directed to recognize the N-terminal HTT regions (summarized in Table 3 and 4; Weiss et al., 2009; Weiss et al., 2011; Macdonald et al., 2014), we have identified two pairs of antibodies which recognize long HTT fragments. Furthermore, we were able to find optimal working conditions for our assays (as indicated by the hook points) and to demonstrate the high reproducibility of the assay, conforming to industry standards. LoD and LLoQ of the assays were 1 and 3 pm with optimal CV values of <7% for intra-assay reproducibility and <20% for interassay reproducibility. Implementation of several full-length purified HTT protein standards in the assays not only allowed us to validate the technical characteristics of the assays and their suitability to quantify HTT in human postmortem brain tissue and human iPSC-derived neural progenitors, but also helped us to demonstrate the importance of using appropriate calibrators matching the polyQ length to be analyzed in the biological samples. It is clear that, in the absence of calibrators with matching polyQ lengths to the sample, there will be an under- or overestimation of the HTT content. Relative comparisons among samples derived from identical or similar genotypes, such as tissue before and after HTT-lowering treatment, would however not be compromised by the effects of polyQ dependence.

**Table 3.** Available assays for HTT detection

Method	Antibody 1	Antibody 1	Reference
TR-FRET	2B7	MW1	Weiss et al., 2009
TR-FRET duplex	2B7	MW1 MAB2166	Weiss et al., 2011
TR-FRET	2B7 2B7	Nov1 MAB2166	Liang et al., 2014
ELISA	HDB4E10	EP867Y	Massai et al., 2013
MesoScale Discovery	CHDI-90000146 CHDI-90000146 CHDI-90000146 CHDI-90000147	MW1 MAB2166 CHDI-90000137 MAB2166	Macdonald et al., 2014
Single-molecule counting	2B7 MW1	MW1 pT3	Wild et al., 2015 Fodale et al., 2017 Cariulo et al., 2017
IP-FCS	HDB4E10	MW1	Southwell et al., 2015

**Table 4.** Technical characteristics of assays for HTT detection

Method	LDL/LLoQ	Dynamic range	Specificity	Reproducibility (intra/inter)	Reference
TR-FRET	25 pm	n.a.	Mouse and human mutant HTT	n.a.	Weiss et al., 2009
TR-FRET duplex	n.a.	n.a.	Mouse and human mutant HTTMouse and human total HTT	n.a.	Weiss et al., 2011
TR-FRET	n.a.	0-4 µg of total protein/well	Endogenous mouse HTT	n.a.	Liang et al., 2014
ELISA	27 pm (2.7 fmol/well)	0.15 pM to 30 nM	Full length (human and mouse)	<10/<20%	Massai et al., 2013
MesoScale Discovery	Low/high pM	1.5–2 log units	Human mutant HTTHuman pan HTTHuman exon1-pan HTTMouse HTT	n.a.	Macdonald et al., 2014
Single-molecule counting	Low fm	6.5/16.5–8000 fM	Mutant HTTPhosphorylated HTT	<20%	Wild et al., 2015 Fodale et al., 2017 Cariulo et al., 2017
IP-FCS	16 fm	n.a.	Expanded recombinant HTT (at high concentration also not-expanded)	n.a.	Southwell et al., 2015

To demonstrate that changes in the AlphaLISA signal correspond to changes in HTT, we analyzed brain lysates from the BACHD mouse model crossed with a mouse expressing Cre under the nestin promoter, thus deleting the mutant HTT protein in neural tissue. We detected a near-complete depletion of the polyQ-dependent signal, i.e., elimination of the mutant HTT protein in the brain, with only a partial decrease in the polyQ-independent assay. These changes were not detectable in liver samples from the same animals. These results suggest that our assays could be used to monitor changes in HTT levels in the presence of HTT modifiers. We implemented the AlphaLISA assay for HTT quantification using the 384-well plate format, as we anticipate that in the future, screening studies will be among the experimental designs to use these assays. Indeed, the simple and wash-free steps of this assay make it very suitable for high-throughput screening (HTS), e.g., investigating compounds or molecular mechanisms lowering HTT levels, as also indicated by Z′ values of 0.74 for the polyQ-independent assay and 0.84 for the polyQ-dependent assay. In our study, using neural progenitors treated with siRNA against HTT, we could demonstrate that the assays report changes in HTT signal, distinguishing between siRNAs with different efficiencies and/or allele selectivity. Notably, although not included in this study, the assay can readily be used to test effect of modifiers on cells in culture after lysis with analysis directly on the plate. Previously developed assays have been used to study HTT modifiers in HTS (Baldo et al., 2012); however, these assays were mostly used for monitoring N-terminal fragments of the protein and did not provide any information on the mutant form of the protein. Furthermore, the increased sensitivity of our assay compared to the TR-FRET assay, would allow improved conditions for HTS, enabling us to use small quantities of starting material—a condition that could be crucial when setting up HTS using iPSC-derived neurons or primary cultures. The sensitivity of the assays described in this study is in the low picomolar range and thus does not allow for the measurement of HTT species in CSF samples. Recent methods using SMC immunoassays or an immunoprecipitation assay combined with flow cytometry have reported measurement of mutant or phosphorylated HTT in the CSF of pre-manifest and manifest HD patients in the low femtomolar range (Southwell et al., 2015; Wild et al., 2015; Cariulo et al., 2017).

HTT is a very large protein that contains several sites for posttranslational modifications as well as proteolytic cleavage. Especially in the presence of polyQ elongation, certain posttranslational modifications and the formation of shorter HTT fragments can be critical for disease-related pathology in the brain. Due to the fact that the glutamine expansion occurs in exon 1 and the requirement for proximity for signal intensity in TR-FRET assays, assays based on that technology used antibody pairs binding to N-terminal regions of the protein, making them insensitive to proteolytic cleavage of the protein as long as the N-terminal sequence was maintained in the product. Here, we adapted a different approach where we aimed to detect long fragments of HTT, spanning up to Pro1220, making the assays sensitive to cleavage by proteases. With emerging tools, the AlphaLISA platform has become amenable to multiplexing (Landeck et al., 2016), which will make it possible to develop further HTT assays that can distinguish not only between total and mutant HTT protein but also the fraction of the protein that has been cleaved into shorter fragments.

HTT protein is cleaved at different residues by caspases and other proteases in both physiologic and pathologic conditions (Saudou and Humbert, 2016). In HD, mutant HTT fragments are often found as the main component of intracellular inclusions and are thus considered to be part of the toxic events leading to neuronal degeneration (DiFiglia et al., 1997). For these reasons, interpreting the results obtained from human postmortem brain tissue might be challenging, especially with the possible coexistence of several posttranslational modifications and overlapping pathologic events. In an attempt to illustrate one example of scenario that could happen, we used purified caspase-6 to cleave purified full-length HTT *in vitro*. As expected, we could detect a reduction in the signal in both polyQ-dependent and -independent assays in the samples treated with caspase-6 as in both assays the cleavage site for caspase-6 (at aa 586) is between the bindings sites for acceptor and donor beads. This characteristic opens up the possibility to multiplex the present assay with a second antibody pair (as used in several of the already published assays) targeting the N-terminal region of the HTT protein, therefore providing a unique readout of both noncleaved and total HTT simultaneously in a single assay.

An important step in our analyses was to take advantage of the assays to investigate changes in HTT protein levels in different brain regions in human postmortem brain tissue from HD cases and controls. Only a few studies have previously addressed the question of whether there were changes in HTT levels in brain regions differentially affected by HD. Also only a few studies were able to compare HTT protein levels, due to the difficulties in quantification of techniques such as Western blot, thus mainly studying RNA expression of HTT (Liu et al., 2013; Evers et al., 2015). For this purpose, we processed samples from the striatum, cerebral cortex, and cerebellum from 10 control cases and 17 HD cases. Striatum and cerebral cortex are brain regions highly affected during HD pathogenesis while cerebellum is known to be relatively spared (Vonsattel et al., 1985). Our analysis revealed that in the striatum of HD cases, there are lower levels of total pool of HTT in the cytoplasmic fraction, as measured by the polyQ-independent assay, potentially due to the more highly selective loss of neurons in this region in HD. This is also reflected in the striatal samples from HD cases having increased levels of mutant HTT in the cytoplasmic fraction compared to cortex and cerebellum, when measured with polyQ-dependent assay. Interestingly, mutant HTT levels in the cortex of HD cases were higher when compared to striatum and cerebellum in the cytoplasmic fraction, potentially due to less neurodegeneration compared with the striatum and therefore more surviving neurons with mutant HTT. In fact, it is possible that the signal is higher in the polyQ-dependent assay due to the presence of increased lengths of the expanded polyQ tract in the mutant HTT protein in the cortical samples from HD cases. Previous studies have found that somatic increases of the polyglutamine tract occur in both the striatum and the cerebral cortex in HD with an estimation of 8%–10% of cells in these regions having mutation increases of >20 CAG repeats and 1%–2% of cells with mutation increases of >150 CAG repeats (Kennedy et al., 2003). As the HD cases included in the present analyses have a Vonsattel grade of II–IV, it is likely that cells with the increased mutation have already died in the striatum whereas the cells in the cortex still remain.

In conclusion, HTT quantification using the alphaLISA platform as shown in this study is a very valuable tool in HD research for several reasons. First, and most importantly, the antibodies used in our work are readily accessible to academic and industrial laboratories. Second, the assay platform itself has several key advantages including its simple implementation and readiness for use in studies requiring high throughput. In addition, the fact that it can be applied as a multiplexed assay enables us to further develop the assay to obtain not only total versus mutant HTT levels, but also fraction of the analyte that is cleaved or otherwise posttranslationally modified, e.g., phosphorylated.

## Synthesis

Reviewing Editor: Leonidas Stefanis, Biomedical Research Foundation of the Academy of Athens Decisions are customarily a result of the Reviewing Editor and the peer reviewers coming together and discussing their recommendations until a consensus is reached. When revisions are invited, a fact-based synthesis statement explaining their decision and outlining what is needed to prepare a revision will be listed below. The following reviewer(s) agreed to reveal their identity: Patrick Weydt, Scott Zeitlin.

The reviewers have highlighted the systematic and thorough development of the new alphaLisa methods and their application in various biosamples. The data are clearly present and prove the robustness of the employed assays. Thee is potential for multiplexing and application in a high-throughput fashion. Overall, the authors report a very useful tool for the HD community. It is surprising that the authors chose not to test their methodology on CSF as this bio-compartment is likely one of the most relevant for proving e.g. target engagement in the upcoming mhtt trials. The reason for this omission may be that the sensitivity of the new assay (1-3 pM) may not be sufficient to detect mutant HTT in small amounts of human CSF (10s-100s of fM/sample). Some comment regarding this issue would be quite important.
